# Analysis of Microplastics in Aquatic Shellfish by Pyrolysis–Gas Chromatography/Mass Spectrometry after Alkali Digestion and Solvent Extraction

**DOI:** 10.3390/polym14183888

**Published:** 2022-09-17

**Authors:** Yingying Zhong, Qibei Bao, Lifeng Yuan, Jiawen Liu, Yan Cai, Xianfeng Chen

**Affiliations:** 1 Ningbo Customs Technology Center, Ningbo 315012, China; 2 Ningbo College of Health Sciences, Ningbo 315100, China; 3 College of Biology and Environment, Zhejiang Wanli University, Ningbo 315100, China

**Keywords:** microplastics, alkali digestion, pyrolysis–gas chromatography–mass spectrometry, aquatic shellfish, nylon

## Abstract

Microplastics are harmful to both marine life and humans. Herein, a pyrolysis–gas chromatography–mass spectrometry (Py-GC/MS) technique for the detection of microplastics in aquatic shellfish is demonstrated. The organic matter in aquatic shellfish was removed by alkali digestion. Subsequently, using hexafluoroisopropanol as the extraction solvent, the extraction method was optimized. The influence of the digestion process on the nature of microplastics was investigated by analyzing the samples before and after the alkali treatment via infrared spectrometry, laser particle sizing, and scanning electron microscopy. Spiked recovery experiments and an analysis of actual samples were performed using PA6 and PA66 as analytes. A quantitative analysis of the characteristic ion fragment produced by high-temperature cracking was performed after chromatographic separation and mass spectrometry identification. The linear range of this method for PA6 and PA66 was 2–64 μg. The limits of detection of PA6 and PA66 were 0.2 and 0.6 μg, while the limits of quantitation were 0.6 and 2.0 μg, respectively. Recovery ranged from 74.4 to 101.62%, with a precision of 4.53–7.56%. The results suggest that the Py-GC/MS technique is suitable for the analysis and detection of trace microplastics in aquatic shellfish.

## 1. Introduction

Microplastics have attracted significant research attention since their initial discovery by Thompson et al. in a study on plastic fragments in seawater and marine sediments [1]. The global production of plastics reached 368 million metric tons in 2018, and at least 8 million metric tons of plastic waste enter the oceans annually, causing a considerable pollution problem in marine environments [2,3,4,5]. Microplastics have been found in shellfish [6], fish [7,8], shrimp [9], table salt, and bottled water [10]. Because marine organisms are strongly affected by microplastics in the environment, the consumption of aquatic products, including fish and shellfish, by humans serves as the primary pathway of microplastic ingestion [11,12]. The uptake of microplastics by marine organisms can cause physical injury, oxidative stress, and damage, has impacts on food intake and reproduction, and can even be fatal [9]. Developing techniques for the detection of microplastics is therefore of the utmost importance.

Existing techniques typically observe the size, shape, and color of microplastics by visual inspection under a microscope or stereomicroscope, while the chemical composition is typically determined by micro-Fourier transform infrared (micro-FTIR) spectroscopy, micro-Raman spectroscopy, and scanning electron microscopy–energy-dispersive X-ray spectrometry (SEM-EDS) [13,14,15,16]. Micro-FTIR, which enables the identification of polymeric chemical components by acquiring the IR spectra of analytes within micro-areas of the sample, is the primary technique for the qualitative analysis of microplastics in various environmental media. Micro-Raman spectroscopy is used to determine the chemical composition of microplastics as small as 1 μm by detecting surface-bound functional groups in the sample material. However, the elimination of the fluorescence background in Raman spectra is a major challenge associated with the technique. SEM-EDS can distinguish microplastics that are primarily composed of carbon from inorganic particles by observing the morphological characteristics and elemental composition of the sample surfaces. A qualitative analysis of the abundance of microplastics within shellfish is currently achieved via visual inspection and manual counting [17]; however, this method is time-consuming, tedious, and prone to significant errors, thereby posing difficulties for accurate quantitation.

In the present study, qualitative and quantitative methods of analyzing microplastics in aquatic shellfish were developed using pyrolysis–gas chromatography–mass spectrometry (Py-GC/MS). The organic matrix in shellfish was eliminated by chemical digestion, then the microplastic particles were extracted using hexafluoroisopropanol for Py-GC/MS analysis. Nylon 6 and nylon 66 were selected as representative microscopic plastic materials because they are likely to be present in aquaculture environments due to their widespread use in fishing nets and ropes. Bivalve shellfish are ideal for monitoring microplastic pollution in marine environments due to their limited mobility, strong regionality, high vulnerability to environmental pollution, and ability to take up microplastics through filter feeding. Among the indicator bivalve species, mussels are currently the most commonly used in microplastics monitoring. Therefore, mussels were selected as the representative species to establish methods for detecting the target microplastics, nylon 6 and nylon 66. By investigating the effects of the digestion and extraction processes on microplastics and optimizing the pretreatment method and instrument conditions, the quantitative measurement of microplastics in aquatic shellfish samples was achieved.

## 2. Materials and Methods

### 2.1. Chemicals and Materials

The chemicals used were as follows: potassium hydroxide (KOH, AR grade; Sinopharm Chemical Reagent Co., Ltd., Shanghai, China), hydrogen peroxide (H_2_O_2_, 30%, AR grade; Sinopharm Chemical Reagent Co., Ltd., Shanghai, China), hydrochloric acid (HCl, AR grade; Sinopharm Chemical Reagent Co., Ltd., Shanghai, China), and hexafluoroisopropanol (CAS: 920-66-1, 99.5% purity; Shanghai Aladdin Bio-Chem Technology Co., Ltd., Shanghai, China), with an ultrapure water system (PC1ANRXM1; ELGA LabWater, High Wycombe, UK).

The materials were as follows: commercially available mussels (purchased from Lulin Seafood Market, Ningbo, Zhejiang, China), nylon 6 powder (PA6, 150 mesh; DuPont, Wilmington, DE, USA), nylon 66 powder (PA66, 150 mesh; DuPont, Wilmington, DE, USA), and a stainless-steel sieve (1800 mesh; Shanghai Yanjing Sieve Manufacturing Co., Ltd., Shanghai, China).

### 2.2. Digestion and Extraction of Microplastics

For digestion, the commercially available mussels were dissected to separate the tissue. The mussel tissue was homogenized in a glass homogenizer. Then, the homogenized mussel meat (10.0000 g) was added to a 10% KOH (*m*/*v*) solution (200 mL). The digestion effects of 1 + 1 HCl (*v*/*v*) and 30% H_2_O_2_ solutions were compared using the same KOH method. The solutions were subjected to vortex shaking to ensure dispersion, and the samples were heated to 60 °C for 2 h in an oven. For the redispersion of the solutions, at 30 min intervals during the 2 h the samples were taken out of the oven for vortex shaking. The samples were then filtered through an 1800-mesh stainless-steel sieve, and the contents of the sieve were washed with deionized water.

For extraction, after vacuum filtration to a near-dry state, the sieve was transferred to a glass Petri dish, and its surface was thoroughly washed three times with hexafluoroisopropanol (15 mL) to ensure the complete dissolution of the microplastic particles. The eluent was concentrated to a near-dry state by heating at 65 °C in a test tube, then dissolved in hexafluoroisopropanol (1 mL). Based on the microplastic content of the sample, an appropriate volume of sample solution was obtained and volatilized on a hot plate at 65 °C in a pyrolysis cup to remove the solvent, and the cup was subsequently loaded into a pyrolysis—gas chromatography—mass spectrometer for analysis.

### 2.3. Matrix Digestion Efficiency

#### 2.3.1. Digestion Efficiency

An electronic balance (XS205DU; Mettler Toledo, Zurich, Switzerland), electric thermostatic air-drying oven (DGG-9053AD; Shanghai Sumsung Laboratory Instrument Co., Ltd., Shanghai, China), electric hotplate (C-MAG HP 10; IKA, Staufen, Germany), centrifuge (3-18KS; Sigma, Neustadt, Germany), and vacuum pump (N 816.3 KT.18; KNF, Freiburg, Germany) were employed to measure the weight recovery of the meat digestion procedure. First, a 10% KOH solution (20 mL) was added to the mussel meat samples (1.0000 g). The samples were heated to 30, 40, 50, 60, and 70 °C for 1, 2, 3, 4, and 5 h, respectively, with shaking at 1 h intervals. After treatment, each sample was centrifuged at 4500 rpm for 5 min, and the digestion efficiency (DE) was calculated from the weight of the bottom precipitate using Equation (1):

(1) $$\mathrm{DE}\;(\%)\;=\;100\%\;-\;\frac{m_{1}}{m}\times 100\%,$$

where *m_1_* and *m* are the mass of the sample after and before digestion (g), respectively.

#### 2.3.2. Microplastic Recovery Rate

Separately, a 10 wt% KOH solution (200 mL) was added to samples of PA6 and PA66 (20.0000 g), which were digested at 60 °C for 2 h with shaking performed at 30 min intervals. The solvent was removed by vacuum filtration, and the contents of the stainless-steel sieve were washed with ultrapure water until a neutral pH was reached. The microplastic particles and sieve were then dried in a glass Petri dish at 90 °C. The sieve and glass Petri dish were weighed before and after the addition of microplastic particles, and the microplastic recovery rate (RE) was calculated using Equation (2):

(2) $$\mathrm{RE}\;\%\;=\;\frac{m_{1}-m_{0}}{m}\times 100\%,$$

where *m*_1_ is the total mass of the microplastics, sieve, and Petri dish after drying (g), *m*_0_ is the mass of the sieve and Petri dish before digestion (g), and m is the mass of added microplastics (g).

#### 2.3.3. IR Spectrometry Analysis

Samples of microplastic particles before and after digestion were pressed into KBr disks and analyzed by an infrared spectrometer (Nicolet 6700; Thermo Fisher Scientific, Waltham, MA, USA) with 64 scans at a resolution of 4 cm^−^^1^ within the wavenumber range of 650–4000 cm^−^^1^.

#### 2.3.4. Laser Particle Size Analysis

The particle size distribution of the microplastics before and after digestion was analyzed using a laser particle size analyzer (Helos-Oasis; Sympatec, Clausthal-Zellerfel, Germany) with water as the dispersion medium in a 2 mm cuvette, ultrasonication at 100% power for 60 s and a pause of 5 s, and a stirring speed of 80 rpm.

#### 2.3.5. Scanning Electron Microscope Analysis

The micromorphology of microplastics before and after digestion was observed with a scanning electron microscope (Regulus 8230; Hitachi, Tokyo, Japan), with the powder dispersing and sticking to the conductive tape on the sample holder. After blowing off the excess sample powder on the tape, the sample holder underwent gold-spray treatment.

### 2.4. Chemical Analysis of Microplastics Using Py-GC/MS

#### 2.4.1. Instrument Conditions

For pyrolysis temperature, the microplastic particles before and after digestion were subjected to a thermogravimetric analysis (TGA209F1 thermogravimetric analyzer; Netzsch, Selb, Germany) under N_2_ atmosphere in a temperature range of 25–810 °C at a heating rate of 10.0 °C/min.

Py-GC/MS was performed using a pyrolyzer (PY-2020iD; Frontier Laboratories, Fukushima, Japan) and gas chromatograph with a mass-selective detector (6890N/5975B; Agilent Technologies, Santa Clara, CA, USA). The pyrolysis temperature, time, and interface temperature were 600 °C, 1 min, and 300 °C, respectively. GC was conducted using a DB-5HT capillary column (30 m × 0.25 mm × 0.10 μm) with an initial column temperature of 50 °C, which was maintained for 5 min before the column was heated at a rate of 20 °C/min to 270 °C and maintained for 14 min. The injection port temperature, split ratio, and solvent delay were 300 °C, 20:1, and 0.10 min, respectively. High-purity helium was used as a carrier gas at a flow rate of 1.0 mL/min. Electron impact (EI) MS was conducted with interface, ion source, and quadrupole temperatures of 280, 230, and 150 °C, respectively. Qualitative determination was achieved with full-scan mode over an MS scan range of 29–600 *m*/*z*, while quantitative determination was accomplished by selected ion monitoring (SIM). Table 1 lists the characteristic ions of the two types of microplastics.

#### 2.4.2. Validation of the Method

##### Calibration Curve

Separately, PA6 and PA66 powders (50 mg) were made up to a volume of 25 mL using hexafluoroisopropanol to obtain a mixed solution of 2 mg/mL. Thereafter, an aliquot of the mixed solution (2.5 mL) was drawn and made up to a volume of 25 mL using hexafluoroisopropanol to obtain a standard working solution of 0.2 mg/mL. Standard working solution samples of 10, 20, 40, 80, 160, and 320 μL were drawn and transferred to 50 μL sample cups. When the volume of the solution exceeded that of the pyrolysis cup, the sample cup was heated to 60 °C with a hotplate to allow solvent volatilization, and the solution was added stagewise. A standard series of 2, 4, 8, 16, 32, and 64 μg was ultimately obtained.

##### LOD and LOQ

The limits of detection (LOD) and limits of quantitation (LOQ) of the quantitative ions shown in Table 1 were determined using signal-to-noise (S/N) ratios of ≥3 and ≥10, respectively.

##### Recovery Rates and Precision

Mussel tissue was used as a matrix material in samples with two concentrations (20 and 200 μg/g) to determine the recovery rate and precision of the method. Each concentration was tested six times. The precision was determined by computing the relative standard deviation (RSD%) of the six sets of data. The recovery rate is the ratio of the quantitatively determined concentration to the spiked amount.

##### Interference

The effects of PC and PET were investigated by analyzing mixed standard solutions of PA6 and PA66 with 1- and 10-fold contents (20 and 200 μg/g, respectively) of PC and PET to obtain selected ion chromatograms of PA6 and PA66.

### 2.5. Statistical Analysis

All statistical analyses were performed using SPSS 19.0 software (IBM Corp., Armonk, NY, USA). All figures were plotted using OriginLab 2021b software (OriginLab Cor., Northampton, MA, USA).

### 2.6. Quality Assurance and Control

To reduce background pollution from the environment, contact between the plastics and the equipment used in the experiment was avoided as much as possible. In addition, the ultrapure water was filtered using a stainless-steel sieve. The iodine flasks, glass Petri dishes, glass test tubes, and glass droppers were washed three times with ultrapure water and oven-dried before use. During sample digestion with a KOH solution, aluminum foil was used to prevent contact between the iodine flask and the stopper. Blank samples were examined prior to each experiment to ensure that the experimental conditions were free from contamination. The sample cups for thermal lysimetry needed to be sterilized at a high temperature before use, and a blank experiment was performed to prevent contamination from entering the experimental field.

## 3. Results

### 3.1. Optimization of Digestion Conditions

**Figure 1:**
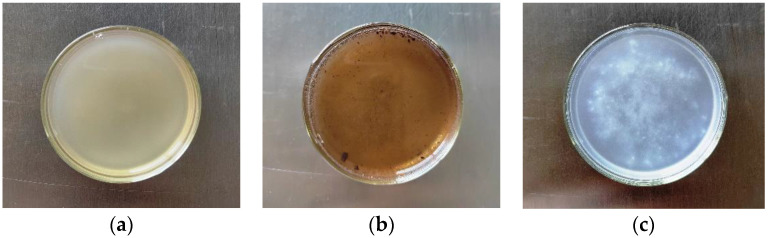
Mussel samples after treatment using three digestion agents: (**a**) 10% KOH; (**b**) 1 + 1 HCl; (**c**) 30% H_2_O_2_.

Chemical methods for extracting microplastics from biological tissue include digestion by an alkali [18], acid [19,20,21], or a strong oxidant [22]. In the present study, we compared the digestion of samples by 10% KOH, 1 + 1 HCl (*v*/*v*), and 30% H_2_O_2_ solutions. At 60 °C, the mussel meat was most rapidly digested by the 10% KOH solution, forming a thick fleshy-pink solution (Figure 1a). After treatment with the 1 + 1 HCl solution, the sample solution turned black, and large pieces of partially digested mussel meat were observed at the bottom (Figure 1b). After treatment with the 30% H_2_O_2_ solution, a milky white suspension presented, and mussel meat fragments were present at the bottom, indicative of incomplete digestion (Figure 1c). Based on these results, 10% KOH was used as the digestion agent for subsequent experiments to optimize the digestion conditions. Masiá et al. observed complete digestion using 200 mL of H_2_O_2_ per 10 g of tissue at 65 °C for 24 h [10]. Ohtani et al. employed a similar digestion method, adding 10 mL of 10 M NaOH (10 g) to samples (10 g) at 60 °C for 48 h [23]. The method described herein requires a significantly shorter digestion time than the other methods, without affecting the Py-GC/MS analysis of microplastics.

**Figure 2:**
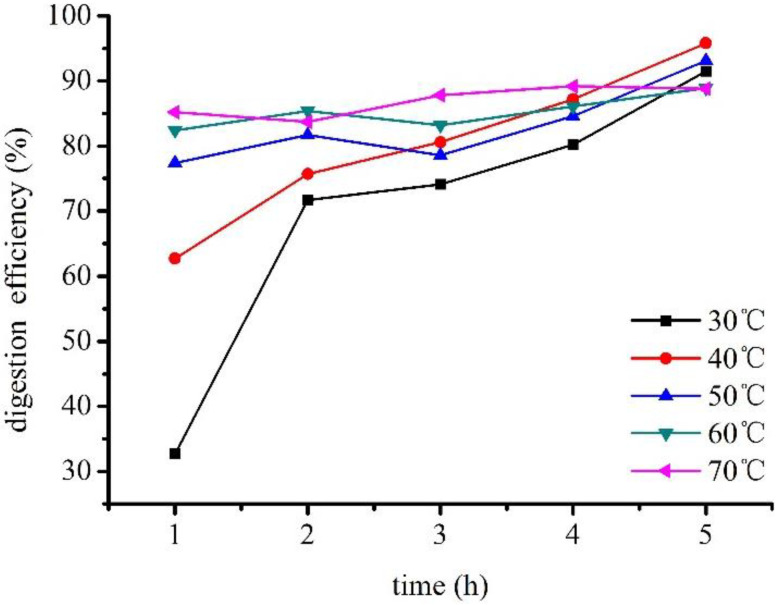
Digestion efficiency of mussels at different digestion temperatures and times.

Digestion efficiency (DE) increased with increasing temperature (Figure 2). At temperatures higher than 50 °C, DE remained essentially stable and did not increase significantly with temperature. With increased digestion time, DE significantly increased at lower temperatures. In particular, DE increased linearly with digestion time at 30 °C. However, increased temperature enabled the digestion of mussel meat within a short time, resulting in a smaller increase in DE. Considering both experimental efficiency and DE, the optimal digestion temperature and time were 60 °C and 2 h, respectively.

### 3.2. Effects of Digestion on Microplastics

The effects of the digestion process on microplastic particles were investigated under the optimal digestion conditions. Nylon is a polyamide resin that is susceptible to hydrolysis under different conditions owing to the large number of amide groups along its main polymer chain. Therefore, the effects of digestion were explored to prevent the loss of microplastic particles during pretreatment, which would affect the accuracy of the experimental results. Equation (2) gives the average RE values of PA6 and PA66 as 98.7 and 97.2%, respectively (*n* = 6), demonstrating that they exhibit essentially no loss of mass after digestion with 10 wt% KOH at 60 °C for 2 h.

**Figure 3:**
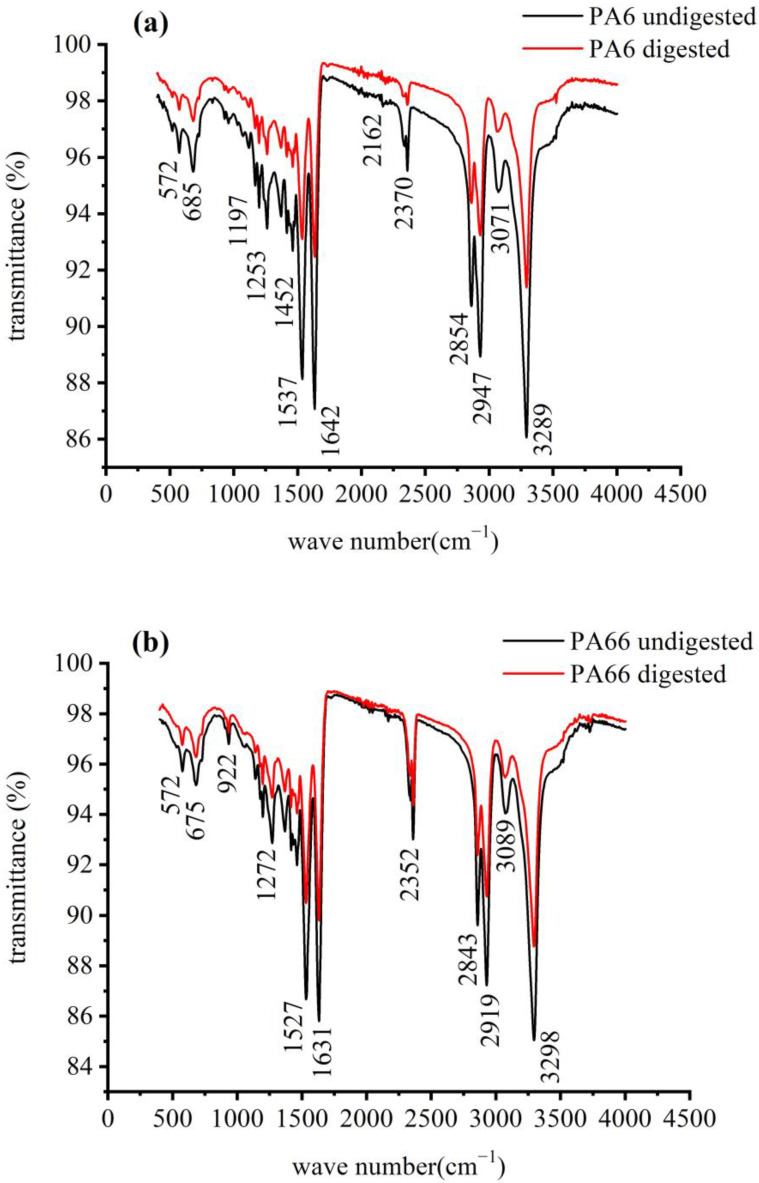
IR spectra of microplastics before and after digestion: (**a**) PA6; (**b**) PA66.

The structural and morphological changes of microplastics were characterized using IR spectroscopy and laser particle size analysis, respectively. The IR spectra of PA6 and PA66 exhibited only slight changes after KOH treatment, with the main characteristic peaks remaining unchanged (Figure 3). For instance, the absorption peaks of the amide groups at 1542 and 1638 cm^−1^, amine groups at 3300 cm^−1^, and methylene groups at 3089, 3071, 2947, 2919, 2854, and 2843 cm^−1^ were identical in the spectra obtained before and after digestion. The intensity of certain absorption peaks decreased after digestion, which may be ascribed to damage to or degradation of certain microplastic structures, such as the rearrangement or aggregation of polymeric chains. The IR spectra of the microplastics showed no significant changes in the main characteristic peak shapes and fingerprint peaks after digestion with a KOH solution for 2 h. This allowed the microplastic materials to be identified by comparing their spectra with those from standard spectral databases.

**Figure 4:**
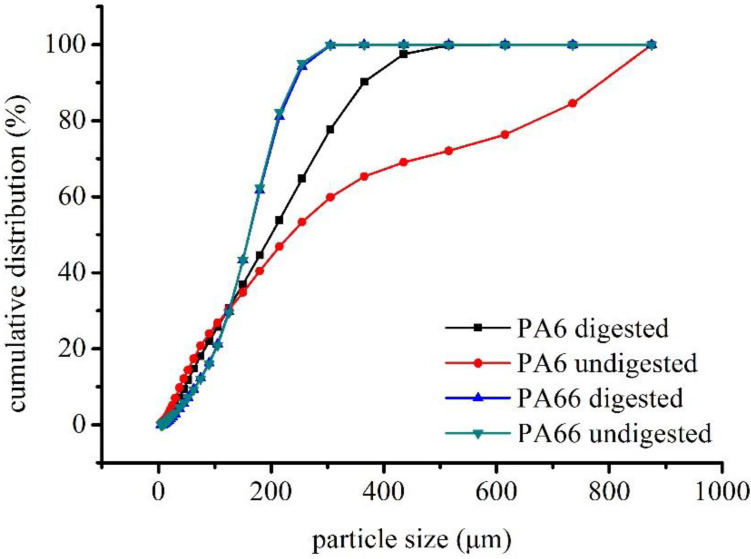
Cumulative particle size distribution of PA6 and PA66 before and after digestion.

A comparison of the particle sizes of microplastics before and after digestion showed that PA6 particles became smaller, whereas PA66 particles remained essentially unchanged after digestion (Figure 4). Large particles were partly degraded into smaller particles after KOH digestion; however, small microplastic particles were not significantly affected (Figure 4). The reduced median particle size and volume mean diameter (VMD) of PA6 are shown in Table 2, along with the increased surface mean diameter (SMD).

**Figure 5:**
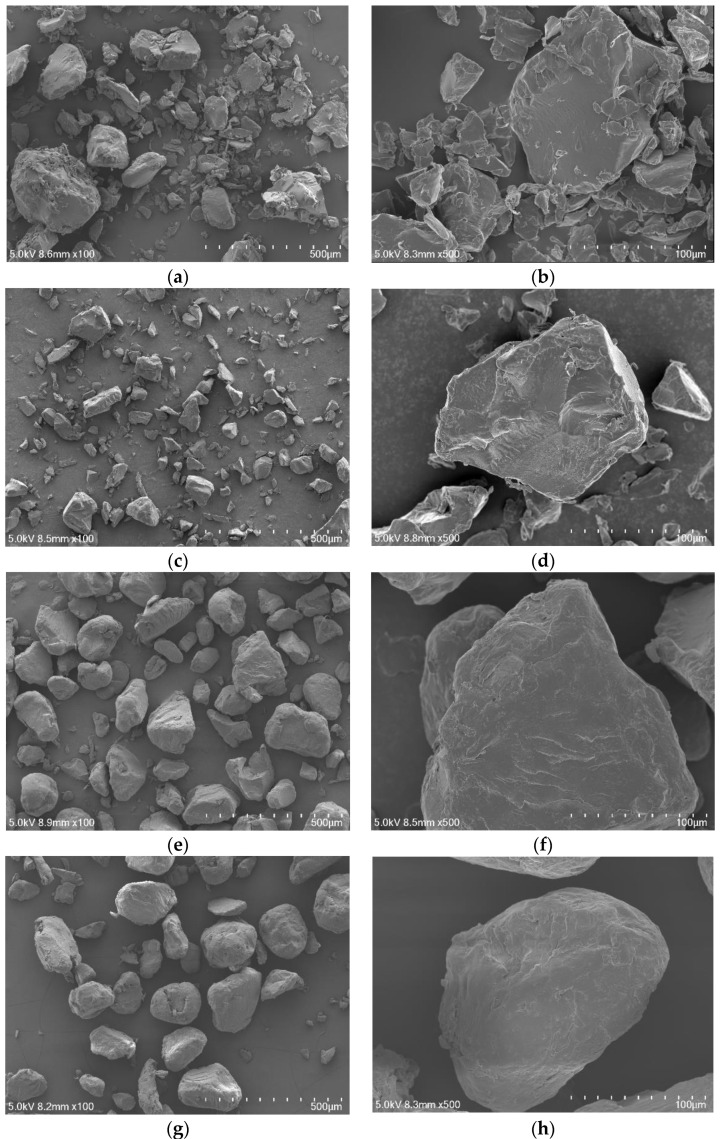
SEM images of microplastics before and after alkali digestion at 100× and 500×: (**a**,**b**) undigested PA6; (**c**,**d**) digested PA6; (**e**,**f**) undigested PA66; (**g**,**h**) digested PA66.

It can be seen in the scanning electron microscope images that the surface morphology of the PA6 and PA66 microplastic particles before and after treatment did not change significantly. Large particles in PA6 powder decreased slightly (Figure 5a,c), as did the particle size, because the polymer chain was partially broken under the action of the alkali solution. However, the morphology and smoothness of the particle surface did not change much (Figure 5b,d). PA66 showed little change in particle size and morphology before and after digestion (Figure 5e,g). Particles after digestion were smoother and rounder than those before digestion. It is possible that sharp parts of particles were eliminated under the action of the alkali solution (Figure 5f,h).

### 3.3. Selection of Pyrolysis Temperature

**Figure 6:**
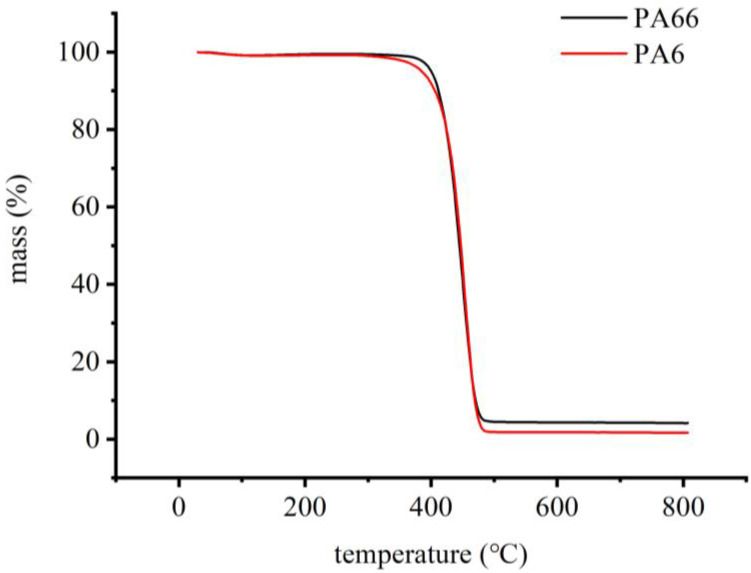
Thermogravimetric analysis of PA6 and PA66.

The pyrolysis temperature affects the generation and distribution of pyrolysis polymer products and is therefore critical for acquiring pyrolysis chromatograms with appropriate characteristics. To ensure the rapid and complete pyrolysis of particles in the pyrolyzer, the degradation of PA6 and PA66 was essentially achieved between 400 and 500 °C (Figure 6). Therefore, 500 °C was selected as the minimum operating temperature of the pyrolyzer to ensure the rapid pyrolysis of microplastic particles and to reduce chromatographic peak tailing.

**Figure 7:**
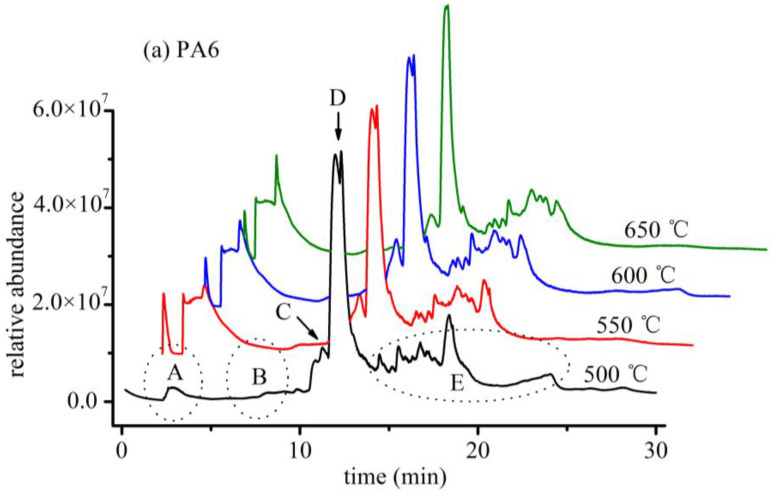

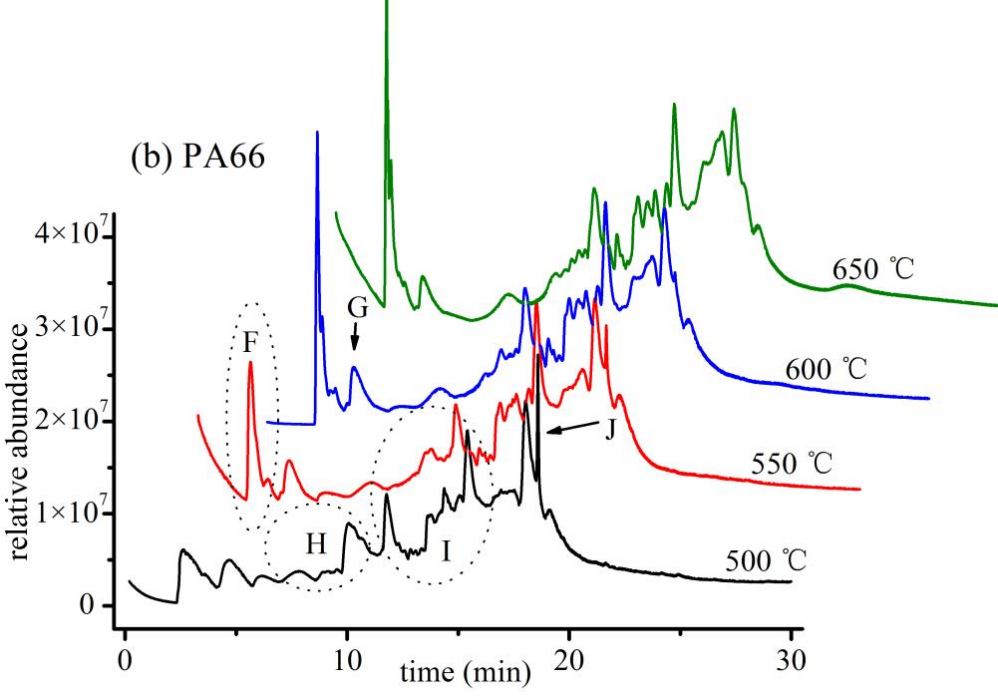
Total ion flow of microplastics by Py-GC/MS at different temperatures for thermal cracking: (**a**) PA6; (**b**) PA66. Region A represents carbon dioxide and propylene; region B represents 1-pentenenitrile and hexanenitrile; region C represents 6-aminohexanenitrile; region D represents caprolactam; region E represents various amides; region F represents cyclopentanone; region G represents 5-hexenamine; region H represents amines, such as 1-hexylamine and 1,6-hexanediamine; region I represents amides; and region J represents 1, 8-diazacyclotetradecane-2, 7-dione.

The cracking temperatures of nylon 6 and nylon 66 gradually increased from 500 to 650 °C, and the cracking of plastic particles changed as the cracking temperature increased (Figure 7). As seen in Figure 6, the cracking of plastic particles changed as the temperature increased, and in general, more fragmented ions of small molecules were produced with increasing temperature, causing increased intensity of some characteristic peaks in response; however, other peaks disappeared owing to high-temperature cracking. A cracking temperature of 600 °C was chosen after comparing the cleavage chromatograms of the two substances at various temperatures.

Region A of the cleavage chromatogram of PA6 shows the presence of small molecule substances such as carbon dioxide and propylene (Figure 7a), and the number of small molecule fragments increased with rising temperature. Region B of the chromatogram, from 7.8 to 9.5 min, mainly shows the presence of nitrile compounds, including 1-pentenenitrile and hexanenitrile, which can be formed by the dehydration of amide bonds during cleavage. The substance appearing at 11.3 min (region C) was identified as 6-aminohexanenitrile. The intensity of this peak increased with temperature, demonstrating that the cleavage of plastic particles was more complete at higher temperatures. The chromatographic peak in region D was identified as the most important cleavage product of PA6, caprolactam, which shows bifurcation at lower temperatures, likely because the cleavage of plastic particles is delayed at low temperatures. This observation is supported by the fact that peak bifurcation decreases and peak shape becomes sharper with increasing temperature. The peaks in region E correspond to various amides produced by the breakage of carbon and nitrogen single bonds on amide bonds, which produces carbonyl and amino radicals. These radicals attack the carbon and nitrogen atoms of other amide bonds, resulting in the formation of a large number of small-molecule amides, the most important and characteristic of which is caprolactam.

The peak area in region F in the cleavage chromatogram of PA66 is very small in the low-temperature section and gradually increases with rising temperature (Figure 7b). The peaks in this region represent small molecules, including cyclopentanone, which is obtained by the cleavage of the amide bond of PA66 to produce amino and carbonyl radicals by cyclization. Region G, the area of which gradually increases with temperature, represents 5-hexenamine, while region H shows amines, including 1-hexylamine and 1,6-hexanediamine, at approximately 7.8 and 10.2 min, respectively, which are formed by the transfer of hydrogen to amino radicals from adjacent carbon atoms. With increasing temperature, the amines become unstable, and the peak area decreases. Region I, from 11.8 to 16.8 min, is dominated by amides. These substances become more numerous with increasing temperature, and thus the intensity of the peaks also increases. Region J represents 1, 8-diazacyclotetradecane-2, 7-dione. The peak area in this region decreases with increasing temperature and is masked by the large amount of amides produced.

After comparing the cleavage chromatograms of the two substances at different temperatures, 600 °C was chosen as the cracking temperature.

### 3.4. Selection of Characteristic Peaks for Quantitative Analysis

**Figure 8:**
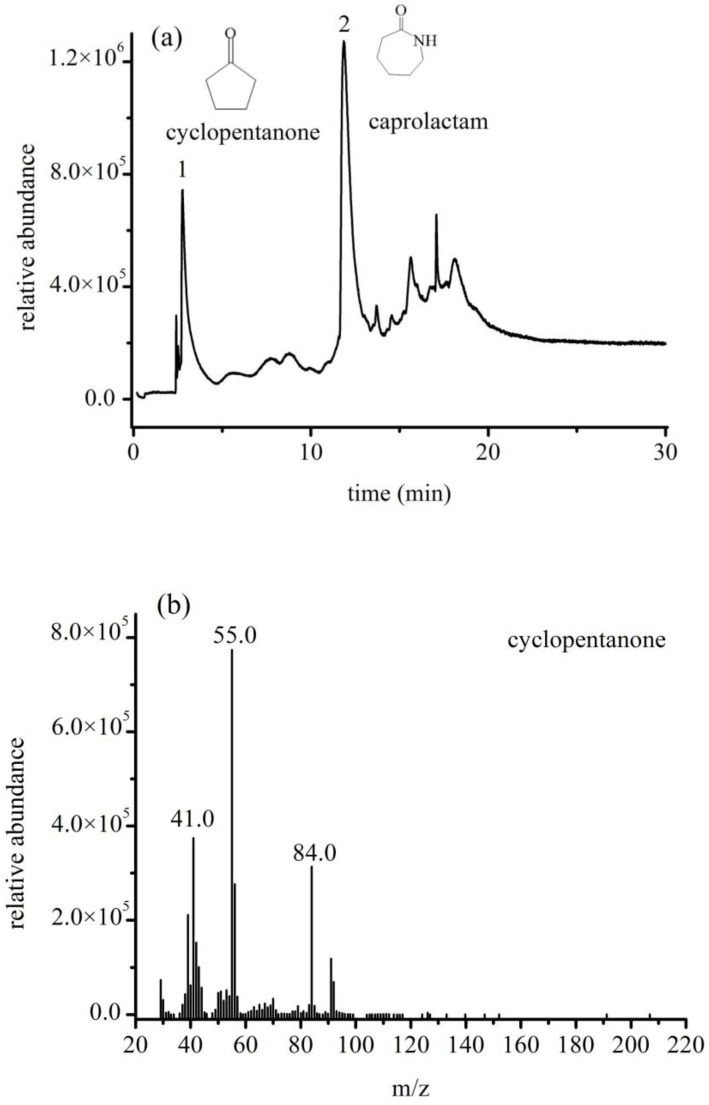

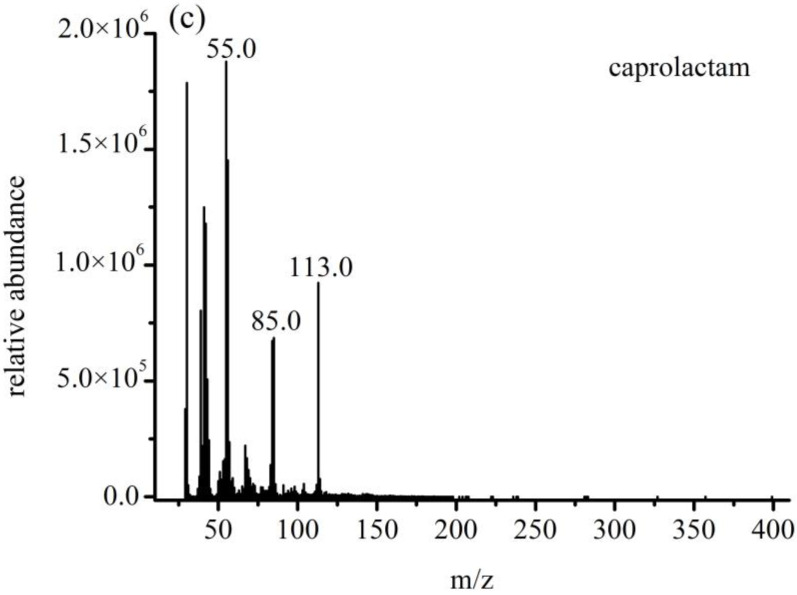
(**a**) Total ion chromatogram (TIC); (**b**) mass spectra of cyclopentanone obtained from Py-GC/MS of PA66; (**c**) mass spectra of caprolactam obtained from Py-GC/MS of PA6. Peaks 1 and 2 in TIC represent cyclopentanone and caprolactam, respectively.

Under the pyrolysis conditions described in Section 2.4.1, a mixture of PA6 and PA66 particles was examined, and the total ion chromatograms and mass spectra of the characteristic pyrolysis products were obtained (Figure 8). The mass spectra show quantitative ions for PA66 and PA6 at *m*/*z* 84 and 113, respectively.

### 3.5. Interference Experiment

**Figure 9:**
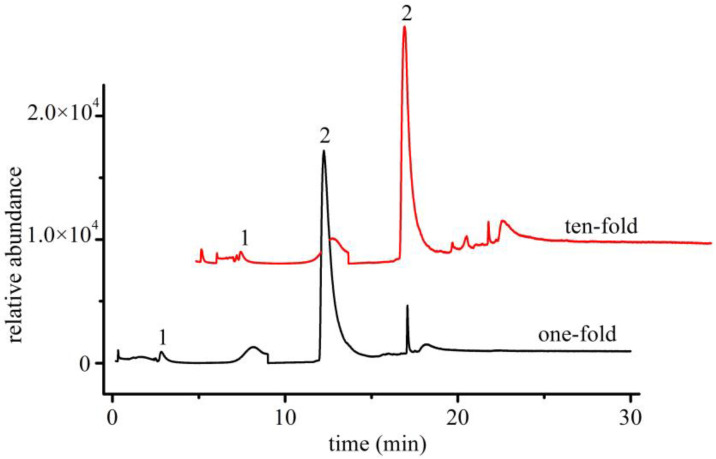
One- and ten-fold interference of PC and PET in PA66 and PA6 standard solutions (1: cyclopentanone; 2: caprolactam).

Because pyrolysis breaks down plastic particles into smaller molecules, identical fragment molecules can be produced by different types of plastics. To avoid interference from other types of microplastics, the solubility of different polymers in the hexafluoroisopropanol extraction solvent was examined. Polycarbonate (PC) and polyethylene terephthalate (PET) were found to be soluble in hexafluoroisopropanol, whereas polystyrene (PS), polypropylene (PP), and polyethylene (PE) were not. Therefore, PS, PP, and PE microplastics were not expected to cause interference in the Py-GC/MS results. The effects of PC and PET were further investigated by analyzing the selected ion chromatograms of PA6 and PA66 obtained from mixed standard solutions with 1- and 10-fold contents of PC and PET (Figure 9). No significant changes in the peak areas or retention times of PA6 and PA66 before or after the addition of PC and PET were observed; thus, it was concluded that the presence of PC and PET particles does not affect the Py-GC/MS results under optimized conditions with a hexafluoroisopropanol extraction solvent.

### 3.6. Testing of Actual Samples

Standard curves were constructed using the method described in Section 2.4.2. Linear regression equations were derived from plots of the mass of the tested substance (X, μg) against the peak area of the quantitative ion (Y). Table 3 lists the various parameters derived for both types of microplastics. The sample load for Py-GC/MS is typically within the range of 10–100 μg [24]. Our experimental results revealed that an increase in the highest point of the standard curve to 128 μg resulted in poorer linearity of the standard curve, indicating that a sample load of 128 μg exceeded the detection capacity of the instrument. Therefore, the upper limit of the linear range was confirmed to be 64 μg.

**Figure 10:**
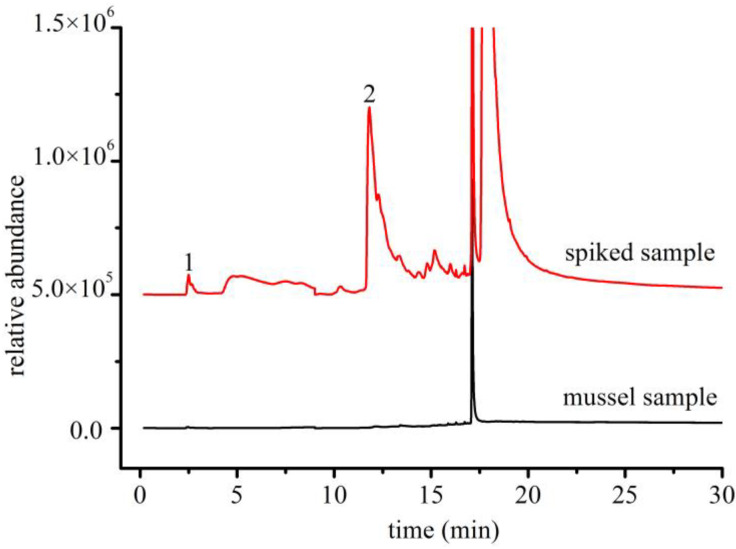
Selected ion chromatograms of mussel samples (concentration, 20 μg/g) (1: cyclopentanone; 2: caprolactam).

Commercially available mussel products were used as samples for the recovery experiment at two concentration levels (Figure 10), with testing performed six times for each concentration. For sample detection, 10 g of mussel was weighed, and the detection and quantification limits were calculated. The detection limits were 0.02 and 0.06 μg/g and the limits of quantitation were 0.06 and 0.20 μg/g for PA6 and PA66, respectively. As this method adopted extraction and enrichment after digestion, the detection and quantitation limits can be further reduced by increasing the sample quantity.

## 4. Discussion

PA6 and PA66 showed essentially no loss of mass after digestion with 10 wt% KOH at 60 °C for 2 h. The changes in particle size after digestion had little effect on the Py-GC/MS results because of the need to dissolve the microplastic particles in a solvent for analysis. Moreover, sieves with appropriate mesh sizes must be selected based on the particle size range to avoid the loss of microplastics during the experimental process. The fact that hexafluoroisopropanol is a suitable solvent to dissolve nylon suggests that using it for the selective extraction of microplastics can allow a more straightforward quantification of PA6 and PA66. Py-GC/MS can directly quantify the weight of microplastics, even within nanoplastic, but if the proper extraction of target microplastics is possible, its ability to extract microplastics in seafood has not yet been reported.

In Py-GC/MS, the pyrolyzer enables the instantaneous degradation of polymers into small molecules that are subsequently injected into the separation column. The pyrolysis products are separated, and the pyrolysis chromatogram is then recorded. The pyrolysis of a singular polymer typically produces extremely complex components that reflect the structure of the original sample. However, chromatograms of mixed samples typically suffer from interference owing to the high number of components. Therefore, the small molecules characteristic of each type of polymer must be identified to achieve accurate qualitative and quantitative detection. Nylon 6 is produced by the polymerization of caprolactam, whereas nylon 66 is formed by the condensation polymerization of adipic acid and hexamethylenediamine. Amide and carbon–nitrogen bonds are prone to breakage at high temperatures because carbon–heteroatom single bonds are weaker than carbon–carbon single bonds [23,24]. Caprolactam is the main pyrolysis product of PA6; other products include carbon dioxide, nitriles, and cyano-group-containing dimers and polymers. The breakage of amide bonds in PA66 produces amine and carbonyl free radicals, with the latter forming cyclopentanone via a cyclization reaction. The pyrolysis products of PA66 therefore mainly consist of cyclopentanone; other pyrolysis products include adiponitrile, 1-hexene, and cyano-group-containing dimers and trimers. In the present study, caprolactam and cyclopentanone were selected as characteristic pyrolysis products of PA6 and PA66, respectively. These observations are in good agreement with those of Anuar et al. [25].

Maurits et al., in their study, measured microplastic levels in sea mussels of less than 20 μg/g [26]. Herein, PA6 and PA66 contents of 0.48 and 0.25 μg/g, respectively, were observed, with recovery rates ranging from 74.4 to 101.62% and relative standard deviations (RSD) ranging from 4.53 to 7.56% (Table 4). Py-GC/MS is mainly employed to analyze the levels of microplastics in environmental samples, including wastewater, soil, and beach sand [27,28,29,30,31,32]. Our method yields low LOD and LOQ, along with high recovery rates and precision, and is therefore suitable for the detection of PA6 and PA66 microplastics in aquatic shellfish samples. It provides a methodological reference for the development of detection methods for other types of microplastics in aquatic shellfish samples. We will continue to investigate the abundance of nylon and nylon 66 in shellfish in the estuary using the optimized method in this paper to assess the consumption risk of microplastics in shellfish in the future.

## 5. Conclusions

A Py-GC/MS method for qualitative and quantitative detection of PA6 and PA66 in aquatic shellfish was demonstrated based on pretreating samples with alkali digestion. Py-GC/MS provides far more accurate mass concentrations than visual inspection, which is currently the method used to determine microplastic particle numbers and concentrations, and can thus facilitate improved data analysis and comparison. In addition, inaccuracies in the results arising from the inadvertent omission of particles during visual inspection can be prevented. The linear range of this method for PA6 and PA66 was found to be 2–64 μg. The limits of detection were 0.2 and 0.6 μg, and the limits of quantitation were 0.6 and 2.0 μg for PA6 and PA66, respectively. Recovery was in the range of 74.4–101.62%, with precision in the range of 4.53–7.56%. The method for the qualitative and quantitative detection of PA6 and PA66 developed in this study can be made applicable to other types of microplastics (PC, PET, PVC, PE, PP, PS, etc.) by altering the testing conditions. This study can serve as a reference for the formulation of standard methods for detecting microplastics in seafood.

## Figures and Tables

**Table 1:** Microplastic ion characteristics.

Microplastic	Characteristic Compound	Retention Time (min)	Characteristic Ions (*m*/*z*)	Abundance Ratio of Ions	Quantitative Ions (*m*/*z*)
PA66	Cyclopentanone	2.654	55:41:84	100:50:40	84
PA6	Caprolactam	11.997	55:113:85	100:50:40	113

**Table 2:** Comparison of particle sizes of PA6 and PA66 before and after digestion.

Microplastic	Median Particle Size, X50 (μm)		Surface Mean Diameter, SMD (μm)		Volume Mean Diameter, VMD (μm)	
	Before Digestion	After Digestion	Before Digestion	After Digestion	Before Digestion	After Digestion
PA6	234.56	200.25	83.26	104.55	330.88	204.71
PA66	160.56	160.91	107.68	110.86	156.97	158.00

**Table 3:** Quantitative parameters for PA6 and PA66.

Microplastic	Linear Equation	Linear Range (μg)	R^2^	LOD (μg)	LOQ (μg)
PA6	Y = 1060000X + 632015	2–64	0.9998	0.2	0.6
PA66	Y = 9670.2X − 44937	2–64	0.9985	0.6	2.0

**Table 4:** Average recovery and RSD for mussel samples (*n* = 6).

Analyte	Background	Low Concentration			High Concentration		
	μg/g	Concentration (μg/g)	Average Recovery Rate (%)	RSD (%)	Concentration (μg/g)	Average Recovery Rate (%)	RSD (%)
PA6	0.48	20	81.5	7.23	200	84.6	5.12
PA66	0.25	20	87.1	7.56	200	94.5	4.53

## Data Availability

The data presented in this study are available on request from the corresponding author.

## References

[1] Thompson R.C., Olsen Y., Mitchell R.P., Davis A., Rowland S.J., John A.W.G., McGonigle D., Russell A.E. (2004). Lost at sea: Where is all the plastic?. Science.

[2] Song X.C., Zhuang W., Cui H.Z., Liu M., Gao T., Li A., Gao Z.H. (2022). Interactions of microplastics with organic, inorganic and bio-pollutants and the ecotoxicological effects on terrestrial and aquatic organisms. Sci. Total Environ..

[3] Xiang Y.J., Jiang L., Zhou Y.Y., Luo Z., Zhi D., Yang J., Lam S.S. (2022). Microplastics and environmental pollutants: Key interaction and toxicology in aquatic and soil environments. J. Hazard Mater..

[4] Mishra S., Singh R.P., Rout P.K., Das A.P. (2022). Membrane Bioreactor (MBR) as an Advanced Wastewater Treatment Technology for Removal of Synthetic Microplastics. Development in Wastewater Treatment Research and Processes, Removal of Emerging Contaminants from Wastewater Through Bio-Nanotechnology.

[5] Cole M., Galloway T.S. (2015). Ingestion of nanoplastics and microplastics by Pacific oyster larvae. Environ. Sci. Technol..

[6] Jahromi F.A., Keshavarzi B.B., Moore F., Abbasi S., Busquets R., Hooda P.S., Jaafarzadeh N. (2021). Source and risk assessment of heavy metals and microplastics in bivalves and coastal sediments of the northern Persian Gulf, Hormogzan Province. Environ. Res..

[7] Chen Y., Shen Z., Li G., Wang K., Cai X., Xiong X., Wu C. (2022). Factors affecting microplastic accumulation by wild fish: A case study in the Nandu River, South China. Sci. Total Environ..

[8] Garcés-Ordóñez O., Saldarriaga-Vélez J.F., Espinosa-Díaz L.F., Patiño A.D., Cusba J., Canals M., Mejía-Esquivia K., Fragozo-Velásquez L., Sáenz-Arias S., Córdoba-Meza T. (2022). Microplastic pollution in water, sediments and commercial fish species from Ciénaga Grande de Santa Marta lagoon complex, Colombian Caribbean. Sci. Total Environ..

[9] Diepens N.J., Koelmans A.A. (2018). Accumulation of plastic debris and associated contaminants in aquatic food webs. Environ. Sci. Technol..

[10] Masiá P., Ardura A., Garcia-Vazquez E. (2022). Microplastics in seafood: Relative input of Mytilus galloprovincialis and table salt in mussel dishes. Food Res. Int..

[11] Ebrahimi P., Abbasi S., Pashaei R., Bogusz A., Oleszczuk P. (2022). Investigating impact of physicochemical properties of microplastics on human health: A short bibliometric analysis and review. Chemosphere..

[12] Mistri M., Sfriso A.A., Casoni E., Nicoli M., Vaccaro C., Munari C. (2022). Microplastic accumulation in commercial fish from the Adriatic Sea. Mar. Pollut Bull..

[13] Käppler A., Fischer M., Scholz-Böttcher B.M., Oberbeckmann S., Labrenz M., Fischer D., Eichhorn K.J., Voit B. (2018). Comparison of μ-ATR-FTIR spectroscopy and py-GCMS as identification tools for microplastic particles and fibers isolated from river sediments. Anal. Bioanal Chem..

[14] Laptenok S.P., Martin C., Genchi L., Duarte C.M., Liberale C. (2020). Stimulated Raman microspectroscopy as a new method to classify microfibers from environmental samples. Environ. Pollut..

[15] de Sá L.C., Oliveira M., Ribeiro F., Rocha T.L., Futter M.N. (2018). Studies of the effects of microplastics on aquatic organisms:what do we know and where should we focus our efforts in the future?. Sci Total Environ..

[16] Hendrickson E., Minor E.C., Schreiner K. (2018). Microplastic abundance and composition in western Lake Superior as determined via microscopy, Pyr-GC/MS, and FTIR. Environ. Sci Technol..

[17] Corami F., Rosso B., Sfriso A.A., Gambaro A., Mistri M., Munari C., Barbante C. (2022). Additives, plasticizers, small microplastics (<100 μm), and other microlitter components in the gastrointestinal tract of com-mercial teleost fish: Method of extraction, purification, quantification, and characterization using Micro-FTIR. Mar. Pollut Bull..

[18] Lopes C., Fernández-González V., Muniategui-Lorenzo S., Caetano M., Raimundo J. (2022). Improved methodology for microplastic extraction from gastrointestinal tracts of fat fish species. Mar. Pollut. Bull..

[19] Cashman M.A., Langknecht T., El Khatib D., Burgess R.M., Boving T.B., Robinson S., Ho K.T. (2022). Quantification of microplastics in sediments from Narragansett Bay, Rhode Island USA using a novel isolation and extraction method. Mar. Pollut. Bull..

[20] Monteiro S.S., Pinto da Costa J. (2022). Methods for the extraction of microplastics in complex solid, water and biota samples. Trends Environ. Anal. Chem..

[21] Sridhar A., Kannan D., Kapoor A., Prabhakar S. (2022). Extraction and detection methods of microplastics in food and marine systems: A critical review. Chemosphere.

[22] Corami F., Rosso B., Roman M., Picone M., Gambaro A., Barbante C. (2020). Evidence of small microplastics (<100 μm) ingestion by Pacific oysters (Crassostrea gigas): A novel method of extraction, pu-rification, and analysis using Micro-FTIR. Mar. Pollut. Bull..

[23] Ohtani H., Nagaya T., Sugimura Y., Tsuge S. (1982). Studies on thermal degradation of Alphatic polyamides by pyrolysis-glass capillary gas chromatography. J. Anal. Appl Pyrol..

[24] Tsuge S., Ohtani H., Watanabe C. (2016). Pyrolysis–GC/MS Data Book of Synthetic Polymers.

[25] Anuar S.T., Altarawnah R.S., Mohd Ali A.A., Lee B.Q., Khalik W.M.A.W.M., Yusof K.M.K.K., Ibrahim Y.S. (2022). Utilizing pyrolysis–gas chromatography/mass spectrometry for monitoring and analytical characterization of microplastics in polychaete worms. Polymers.

[26] Halbach M., Vogel M., Tammen J.K., Rüdel H., Koschorreck J., Scholz-Böttcher B.M. (2022). 30 years trends of microplastic pollution: Mass-quantitative analysis of archived mussel samples from the North and Baltic Seas. Sci. Total Environ..

[27] Lou F., Wang J., Sun C., Song J., Wang W., Pan Y., Huang Q., Yan J. (2022). Influence of interaction on accuracy of quantification of mixed microplastics using Py-GC/MS. J. Environ. Chem. Eng..

[28] Matsui K., Ishimura T., Mattonai M., Iwai I., Watanabe A., Teramae N., Ohtani H., Watanabe C. (2020). Identification algorithm for polymer mixtures based on Py-GC/MS and its application for microplastic analysis in environmental samples. J. Anal. Appl. Pyrol..

[29] Roscher L., Halbach M., Nguyen M.T., Hebeler M., Luschtinetz F., Scholz-Böttcher B.M., Primpke S., Gerdts G. (2022). Microplastics in two German wastewater treatment plants: Year-long effluent analysis with FTIR and Py-GC/MS. Sci. Total Environ..

[30] Chouchene K., Nacci T., Modugno F., Castelvetro V., Ksibi M. (2022). Soil contamination by microplastics in relation to local agricultural development as revealed by FTIR, ICP-MS and pyrolysis-GC/MS. Environ. Pollut..

[31] Funck M., Yildirim A., Nickel C., Schram J., Schmidt T.C., Tuerk J. (2020). Identification of microplastics in wastewater after cascade filtration using Pyrolysis-GC–MS. MethodsX.

[32] La Nasa J., Biale G., Mattonai M., Modugno F. (2021). Microwave-assisted solvent extraction and double-shot analytical pyrolysis for the quali-quantitation of plasticizers and microplastics in beach sand samples. J. Hazard Mater..

